# Predicting the Tensile Behaviour of Ultra-High Performance Fibre-Reinforced Concrete from Single-Fibre Pull-Out Tests

**DOI:** 10.3390/ma15145085

**Published:** 2022-07-21

**Authors:** Konstantin Hauch, Kasem Maryamh, Claudia Redenbach, Jürgen Schnell

**Affiliations:** 1 Department of Mathematics, Technische Universität Kaiserslautern, 67663 Kaiserslautern, Germany; redenbach@mathematik.uni-kl.de; 2 Institute of Concrete Structures and Structural Design, Faculty of Civil Engineering, Technische Universität Kaiserslautern, 67663 Kaiserslautern, Germany; kasem.maryamh@bauing.uni-kl.de (K.M.); juergen.schnell@bauing.uni-kl.de (J.S.)

**Keywords:** computed tomography, fibre-reinforced concrete, mechanics, quantitative image analysis, stochastic modelling, stress–strain diagram, tensile tests

## Abstract

In this paper, a prediction model for the tensile behaviour of ultra-high performance fibre-reinforced concrete is proposed. It is based on integrating force contributions of all fibres crossing the crack plane. Piecewise linear models for the force contributions depending on fibre orientation and embedded length are fitted to force–slip curves obtained in single-fibre pull-out tests. Fibre characteristics in the crack are analysed in a micro-computed tomography image of a concrete sample. For more general predictions, a stochastic fibre model with a one-parametric orientation distribution is introduced. Simple estimators for the orientation parameter are presented, which only require fibre orientations in the crack plane. Our prediction method is calibrated to fit experimental tensile curves.

## 1. Introduction

Ultra-high-performance concrete (UHPC) is characterised by a high packing density, which yields beneficial properties such as high compression strength (>150 MPa) and durability [1,2,3,4,5]. To ensure a ductile behaviour under compression, it is indispensable to add steel fibres to UHPC [6,7,8,9,10]. The high bond capacity of the ultra-high performance fibre-reinforced concrete (UHPFRC) results in a reasonably high post-crack tensile strength [11,12]. This enables the use of UHPFRC as integrated material without conventional reinforcement.

The post-crack tensile strength of UHPFRC highly depends on geometric characteristics of the fibre system such as fibre content, shape, aspect ratio, spatial arrangement, and orientation. The latter two may vary depending on production parameters of the concrete [13]. A high tensile strength is obtained if the crack-crossing fibres are evenly distributed over the crack and if the fibres are aligned to the tensile axis [13,14,15].

Several authors [16,17,18,19,20,21,22,23,24,25,26,27] proposed prediction models for the tensile behaviour based on single-fibre pull-out force contributions. The prediction models by Li et al. [16] and Wuest et al. [17] integrate the force contributions of crack-crossing fibres over the entire crack plane in the tensioned fibre reinforced concrete. Such a sectional analysis provides a relation between stress and crack opening. The force contribution of individual crack-crossing fibres can be approximated from single-fibre pull-out tests for selected fibre orientations and embedded lengths [18,21,27,28,29] or by analytical equations [19,20,23,24]. For modelling purposes, the experimental single-fibre pull-out curves are averaged [25] or idealised [23,27] as piecewise linear with cut points equal to the tensile force at the end of the linear phase and the ultimate force. Besides the fibres, also the matrix contributes to the tensile resistance. Experimental fibre-pull-out curves naturally contain information on fibre–matrix bond properties. If such data are not available, the matrix resistance contribution can be modelled by bilinear [30,31], trilinear [32,33], or exponential [22] functions and added to the fibre force contributions.

A common assumption in calculating the force contributions of crack-crossing fibres is that the fibre orientation is uniformly distributed [19,22,31,34,35], i.e., that concrete is an isotropic material—this simplifies calculations. In practice, however, the fibre orientation distribution deviates significantly from this assumption [13]. This should be taken into account for formulating more realistic models. Empirical fibre orientation distributions determined by image analysis are used in [25,27,36] while [37] derive orientation information from hydrodynamic equations related to concrete flow. The authors are not aware of any study that models the fibre direction distribution stochastically beyond the previously mentioned simple uniformity assumptions or empirical investigations.

An alternative prediction approach is to derive simple analytical equations based on concrete characteristics. A review of such models for predicting the shear capacity is given by [38]. None of these take the fibre orientation distribution into account.

Furthermore, (nonlinear) finite element approaches are nowadays popular, for instance, to predict the overall hysteretic response of cyclic-loaded SFRC [39] or to verify the micromechanics-based fibre-bridging curves of tensioned UHPFRC [23]. Drawbacks of FE methods are the higher computational effort compared to analytical methods and that values of material parameters (such as friction parameters) have to be available. Simulating the contact relationship between steel fibre and matrix via contact [40,41] and coupling [42,43] algorithms requires identical locations for fibre and matrix nodes. This is impractical in the mesh generation and for performing numerical calculations incorporating large quantities of small fibres [35]. The recently introduced model of [35] overcomes this problem, but again only considers uniformly distributed fibre orientations.

Combining suitable imaging techniques with quantitative image analysis is an established way to measure geometric characteristics of the fibre system. For the prediction of tensile behaviour, characteristics of fibres intersecting the crack plane are required; therefore, 2D images of cross sections of concrete specimens are frequently studied; however, the fibre orientation can only be roughly determined from such sections [31,44,45], and the measurement of the embedded length is not possible at all [22]. A more accurate characterisation is obtained by quantitative analysis of micro-computed tomography (μCT) images that allow for a reconstruction of the whole fibre system in 3D [46,47,48].

This work presents a tensile prediction model for UHPFRC-specimens based on a stochastic model for the 3D fibre system and an extensive single-fibre pull-out study. Input parameters for the fibre model are the distribution of fibre orientations, the fibre volume fraction, and the dimensions (length, diameter) of the fibres. The fibre orientation distribution is modelled by a one-parametric distribution family whose parameter β controls the fibre anisotropy, i.e., the scatter of orientations about the preferred direction. The parameter can be estimated either from a sample of fibres observed in a 3D specimen (3D case) or from fibres crossing a planar section of the specimen (2D case).

For fitting the fibre model, a UHPFRC-specimen from a previous study [13] was scanned by micro-computed tomography to reconstruct and analyse its 3D fibre system. This way, embedded length and orientation of the fibres intersecting the crack observed in a mechanical test of the specimen could be determined. A model for the single-fibre contributions to the tensile behaviour is obtained by fitting piecewise linear functions to the force–slip curves observed in single-fibre pull-out tests. Following [34,49], the predicted tensile curve is the sum of all single-fibre pull-out force contributions of crack-crossing fibres. In practice, the tensile behaviour of individually embedded fibres (as used in the pull-out tests) may show deviations from the behaviour of fibres in a larger UHPFRC sample [31]. To take this effect into account, scaling and shifting parameters are introduced, which allow for a calibration of the prediction model to stress–strain curves recorded in experimental tensile tests. Randomising some of the model components yields more realistic curve shapes than a deterministic model.

Our main contribution is the introduction of a stochastic fibre model with a fibre orientation distribution that goes beyond the simple assumptions often considered in the literature (all fibres aligned in one orientation or every fibre orientation is equally probable). Furthermore, we discuss the widespread but flawed assumption that the fibre orientation distribution of crack-crossing fibres coincides with that of the entire 3D fibre system. We present correct and easy parameter estimators for our orientation distribution model. Our fibre model serves as an input to the tensile prediction model, allowing us to simulate the stochastic variability and uncertainty in the tensile prediction. Additionally, certain production parameters such as the fibre volume fraction or the orientation distribution can be varied in the model allowing for a prediction even for cases where no experimental data are available. Apart from the randomness of the fibre geometry, we also randomise the model for single-fibre force contributions. This provides a further means of incorporating uncertainty in our prediction approach and results in more realistic shapes of the stress–strain curves.

## 2. Materials and Methods

### 2.1. Production and Characterisation of the UHPFRC-Specimens

We consider a UHPC-mixture with a maximum grain size of 1 mm. Details on the specification can be found in Table 1 (or see the description of mixture M02 in [13]). Concrete was produced by using the Eirich-Intensive Vacuum mixer of 5 L volume capacity. The same procedure and mix regime as given in [13] were applied. The fresh concrete tests showed similar spread flow, bulk density, and void content values as reported in [13]. For reinforcement, fibres with an ultimate tensile strength of 2800 MPa and elasticity modulus of 200,000 MPa were used. The fibre volume fraction ($V_{V}$) was chosen as 2%, and the fibre aspect ratio was $l_{f}/d_{f}$ = 12.5/0.2 [mm/mm]. Specimens of size 40 × 40 × 160 mm${}^{3}$ were produced. The casting of the specimens was carried out from one side of the formwork, reproducing the configuration M02F2s02 from [13]. The specimens were cured in a climate chamber for 28 days.

### 2.2. Imaging and Image Segmentation

Specimen M02F2s02 from [13] was scanned by micro-computed tomography (μCT) at the Fraunhofer Institut für Techno- und Wirtschaftsmathematik in Kaiserslautern, Germany. To reduce grey value variations in the images, the cubic specimen was placed in a cylindrical UHPC shell during the scanning process. The CT tube was a Feinfocus FXE 225.51 with a maximum acceleration voltage of 225 kV and maximum power of 20W. A Perkin Elmer detector XRD 1621 with 2048 × 2048 pixels was used. The tube voltage was 190 kV, the target electricity 65 μA, and the power 12 W. Tomographic reconstructions were obtained from 800 projections. The specimen of size 40 × 40 × 160 mm${}^{3}$ corresponds to a reconstructed image of 441 × 441 × 1766 voxels with a voxel edge length of 90.6 μm. The fibre system was segmented from the grey value image as described in [13].

For calculating the tensile force contribution of single fibres in a loaded composite, the individual fibres have to be separated in the segmentation. Due to the coarse image resolution, the space between touching fibres is not sufficiently resolved. Hence, labelling the connected components of the fibre system leads to the formation of fibre clusters. For separating fibres in the clusters, a particle separation based on the watershed transform was applied [50]. During this procedure, fibres were split into segments, which were merged manually to reconstruct the single fibres.

After CT scanning, a four point bending test was performed on the sample, see [13] for details. During this test, the specimen developed an approximately planar crack parallel to the xy plane in the CT image. The location of this crack was identified in the CT scan. Due to the complexity of the single-fibre segmentation, the analysis was restricted to the vicinity of the crack plane such that orientation and embedded length of all fibres crossing the crack could be determined.

### 2.3. Tensile Tests

Three concrete samples were tested experimentally in uniaxial tensile tests. The tensile test regime is as in [15] and briefly summarised in the following. For testing, the specimen was placed in the gripping jaws with a contact area of 40 × 60 mm${}^{2}$ and fixed by six bolts, which were pulled by a moment of 110 Nm. This pull moment was determined as the maximum such that no cracks occurred in the clamping area. The specimen was fixed in the pull machine by two nuts. In this setup, a field of 40 mm length located in the centre of the specimens was tensioned. The tests were carried out in a displacement-controlled manner. A low load rate of 0.1 mm/min was chosen. The lengthening of the 40 mm field was measured by using two extensometers. The test was stopped as soon as the lengthening of the field reached 2 mm. During the uniaxial tensile tests, load-lengthening curves were recorded.

It is known that eccentricity occurs in the internal resisting forces due to a non-uniform distribution of fibres in the cross-section. Nevertheless, the stress distribution over the cross-section was assumed to remain uniform during the initial cracking phase and after crack localisation. Thus, the uniaxial equivalent stress reads $\sigma =\frac{F}{A}$, where *F* is the force value and *A* is the area of the cross-section. The strain reads $\varepsilon =\frac{\Delta L}{L}$, where the measured lengthening ΔL is divided by the tensioned length *L* = 40 mm.

Two strength values were computed for every specimen: the elastic post-crack tensile strength ($\sigma_{el}$), which corresponds to the force at the end of the linear phase in the curve (limit of proportionality) in sense of [51], and the ultimate post-crack tensile strength ($\sigma_{ult}$), which corresponds to the maximum force reached.

### 2.4. Modelling Single-Fibre Pull-Out Curves

For performing single-fibre pull-out tests, individual fibres were embedded in a concrete slab. The same concrete mixture and fibres as described in Section 2.1 were used. The length $l_{e}$ of the fibre part embedded in the concrete and the inclination angle θ of the fibres with respect to the pull-out direction were varied. The embedded length $l_{e}$ was chosen as one half, one third or one sixth of the fibre length, i.e., $l_{e}$ is $l_{f}/2=6.25$ mm, $l_{f}/3\approx 4.17$ mm or $l_{f}/6\approx 2.08$ mm. The inclination angle θ was varied from 0${}^{\circ }$ to 80${}^{\circ }$ in steps of 10${}^{\circ }$. For testing, the free end of the fibre was clamped between two metal jaws of the testing machine and pulled out while rigidly fixing the concrete slab. During the procedure, force–slip curves reporting the force *P* applied and the slip *s* were recorded. At least six fibres were tested for each combination $\left(\theta ,l_{e}\right)$ resulting in more than 162 single-fibre pull-out curves.

**Figure 1:**
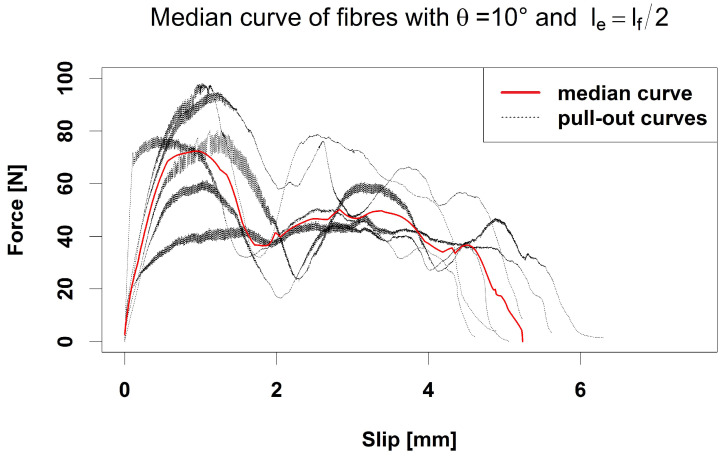
Median curve (red solid) of the single-fibre pull-out curves (black dotted) with angle $\theta =10^{\circ }$ and $l_{e}=l_{f}/2$.

Figure 1 shows the force–slip curves $P_{i}\left(s,\theta ,l_{e}\right),\;i=1,\cdots ,6,$ of the six tested fibres with $\theta =10^{\circ }$ and $l_{e}=l_{f}/2$. We denote by $\dot{P}\left(s,\theta ,l_{e}\right)$ the median curve, which is the point-wise median of the six single-fibre pull-out curves for a fixed combination ($\theta ,l_{e}$), i.e.,

(1) $$\begin{array}{c} \dot{P}\left(s,\theta ,l_{e}\right)=med\left(P_{1}\left(s,\theta ,l_{e}\right),\cdots ,P_{6}\left(s,\theta ,l_{e}\right)\right),\;s\in \left[0,l_{e}\right]. \end{array}$$

**Figure 2:**
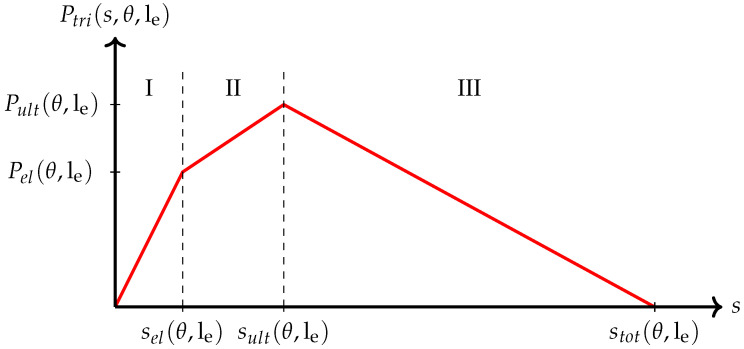
Simplified single-fibre pull-out curve. Phase I and II given as linear increasing branches and phase III given as linear descending branch.

In the next step, we fit a piecewise linear model to the observed force–slip curves. In the literature, several approaches (bilinear, trilinear, exponentially decreasing) for modelling single-fibre pull-out curves have been suggested, see [31]. Based on the results of our single-fibre pull-out tests, we decided to use a three-phase model. Phase I represents the linear elastic part of the curve up to the yield strength $P_{el}$ that is reached at slip $s_{el}$. Phase II is the nonlinear part up to the ultimate force $P_{ult}$ at slip $s_{ult}$. For simplicity, this part is also described by a linear model. Phase III is a linear descending branch up to complete fibre pull-out at slip $s_{tot}$, i.e., the last recorded slip value. See Figure 2 for an illustration of the model.

For fitting the model, the force and slip values derived from the six fibre-pull-out tests per combination $\left(\theta ,l_{e}\right)$ are averaged. We use the median values

$$\begin{array}{ccc} P_{el,med}\left(\theta ,l_{e}\right) & = & med(P_{el,1}\left(\theta ,l_{e}\right),\cdots ,P_{el,6}\left(\theta ,l_{e}\right))\\ P_{ult,med}\left(\theta ,l_{e}\right) & = & med(P_{ult,1}\left(\theta ,l_{e}\right),\cdots ,P_{ult,6}\left(\theta ,l_{e}\right))\\ s_{el,med}\left(\theta ,l_{e}\right) & = & med(s_{el,1}\left(\theta ,l_{e}\right),\cdots ,s_{el,6}\left(\theta ,l_{e}\right))\\ s_{ult,med}\left(\theta ,l_{e}\right) & = & med(s_{ult,1}\left(\theta ,l_{e}\right),\cdots ,s_{ult,6}\left(\theta ,l_{e}\right))\\ s_{tot,med}\left(\theta ,l_{e}\right) & = & med(s_{tot,1}\left(\theta ,l_{e}\right),\cdots ,s_{tot,6}\left(\theta ,l_{e}\right)). \end{array}$$

The numerical values are given in Appendix A, Table A1. When performing a robust two-factor ANOVA, equality of means is rejected for each of the five characteristics. One exception is $P_{ult}$ where the hypothesis of equal means over angles cannot be rejected at the 5% level (*p*-value = 0.083). In spite of this finding, we use individual values for all $\left(\theta ,l_{e}\right)$ groups for all five characteristics rather than merging groups for $P_{ult}$.

In summary, the model reads

(2) $$\begin{array}{c} P_{tri}\left(s,\theta ,l_{e}\right)=\left\{\begin{array}{cc} p_{1}\cdot s & 0\leq s\leq s_{el,med}\left(\theta ,l_{e}\right)\\ \; & (\mathrm{phase}\;I)\\ p_{2}\cdot s+r_{1} & s_{el,med}\left(\theta ,l_{e}\right)\leq s\leq s_{ult,med}\left(\theta ,l_{e}\right)\\ \; & (\mathrm{phase}\;\mathrm{II})\\ p_{3}\cdot s+r_{2} & s_{ult,med}\left(\theta ,l_{e}\right)\leq s\leq s_{tot,med}\left(\theta ,l_{e}\right)\\ \; & (\mathrm{phase}\;\mathrm{III}). \end{array}\right. \end{array}$$

**Figure 3:**
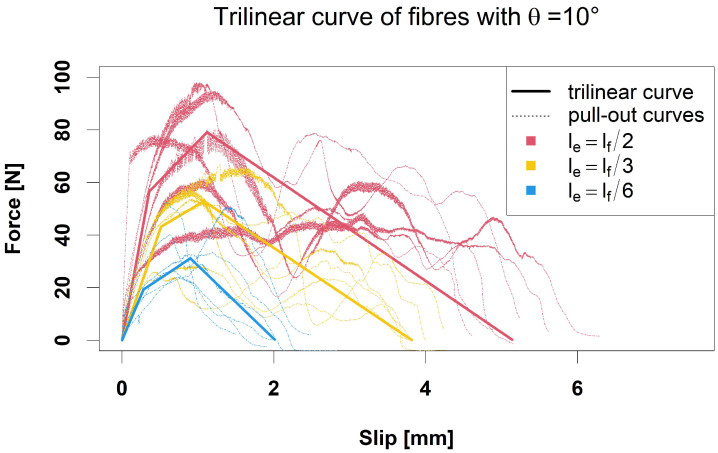
Trilinear curves (solid) of the single-fibre pull-out force curves (dotted) with angle $\theta =10^{\circ }$ and embedded lengths $l_{e}=l_{f}/2$, $l_{e}=l_{f}/3$, $l_{e}=l_{f}/6$.

Formulas for and estimates of the parameters $p_{1},p_{2}>0,p_{3}<0$, and $r_{1},r_{2}\in R$ are summarised in Appendix A, Table A2. Figure 3 illustrates the model for $\theta =10^{\circ }$ and $l_{e}=l_{f}/2,l_{f}/3,l_{f}/6$.

### 2.5. Prediction Model for Tensile Stress

**Figure 4:**
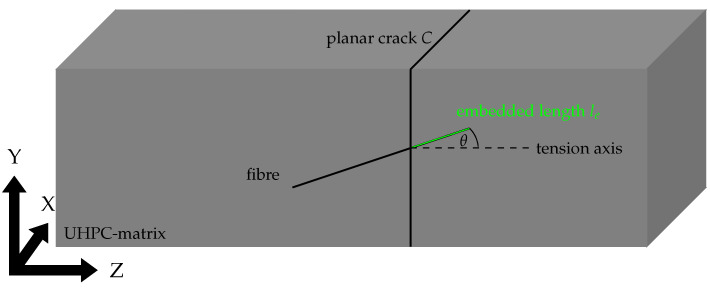
UHPC matrix with one fibre intersecting a planar crack *C* (assumed to be embedded in the (X,Y)-plane) at crack width w=0. The crack divides the fibre in two parts. The embedded length $l_{e}$ is the length of the shorter (green) fibre part. The angle between fibre and tension axis (assumed to be the Z-axis) is denoted by θ.

Our model is based on the following assumptions (see also [16,49]):

- The UHPC matrix is a statistically homogeneous material [52], (p. 28).
- Fibres are straight with cylindrical geometry. The fibres have fixed length $l_{f}$ and diameter $d_{f}$.
- The spatial distribution of the fibre positions in the UHPC is statistically homogeneous [52], (p. 28).
- The fibre orientation is random following a given probability density function (p.d.f.) *p*.
- The specimen is uniaxially strained.
- During loading, the specimen develops a planar crack *C* of width *w* and area $A_{C}$ orthogonal to the tension axis, see Figure 4.
- With growing crack width, the shorter fibre end is pulled out of the concrete matrix. The longer end is not affected.
- The fibres behave linearly elastic.
- The matrix deformation and the Poisson effect of the fibres during pull-out are neglected. The fibre–matrix bond is frictional.

If the strain ε exceeds $\varepsilon_{r}$, the strain at the uniaxial tensile strength of the UHPC, a crack starts to form. The crack width *w* is given by

(3) $$\begin{array}{c} w=\left\{\begin{array}{cc} 0, & \varepsilon \leq \varepsilon_{r}\\ (\varepsilon -\varepsilon_{r})\cdot L, & \varepsilon >\varepsilon_{r} \end{array}\right. \end{array}$$

with L=40 mm and $\varepsilon_{r}=0.00087$. Fibres crossing the crack are divided into the two segments to the left and to the right of the crack plane. We denote the length of the shorter segment by $l_{e}$, see Figure 4, such that $l_{e}\in \left[0,l_{f}/2\right]$. With increasing crack width *w*, the shorter fibre end is pulled out of the concrete until the fibre becomes detached at $w>l_{e}$.

For predicting the stress evolution with increasing crack width *w*, the single-fibre pull-out forces $P(s,\theta ,l_{e})$ of the fibres crossing, the crack have to be taken into account. Due to the assumption that only the shorter fibre end is pulled out of the concrete, the slip *s* corresponds to the crack width *w*. Furthermore, $l_{e}$ in the single-fibre pull-out tests corresponds to the embedded length $l_{e}$. Thus, we use s=w and $l_{e}=l_{e}$ in (2).

Assume that the planar crack *C* is crossed by *N* fibres with given inclination angles and embedded lengths $\left(\theta_{1},l_{e,1}\right),\ldots ,\left(\theta_{N},l_{e,N}\right)$. As experimental single-fibre pull-out curves are only available for a discrete set of θ and $l_{e}$ values, the forces $P(w,\theta ,l_{e})$ of fibres in *C* are binned into classes as given in Table 2.

Fibres crossing the crack counteract the opening of the crack. According to [34,49], the composite stress (or mean resistance force per unit area) at crack width *w* is obtained by

(4) $$\begin{array}{ccc} \sigma_{ct}\left(w\right) & = & \lambda_{c}\int_{0}^{\frac{l_{f}}{2}}\int_{0}^{\frac{\pi }{2}}P\left(w,\theta ,l_{e}\right)p_{c}\left(\theta ,l_{e}\right)\;d\theta \;dl_{e}, \end{array}$$

where $p_{c}\left(\theta ,l_{e}\right)$ denotes the joint probability density of inclination angle and embedded length of crack-crossing fibres. $\lambda_{c}$ is the mean number of fibres per unit area in *C*.

We approximate $\sigma_{ct}\left(w\right)$ by first replacing the integral in (4) by a sum over all fibres observed in the crack. In the second step, angles and embedded lengths are binned such that model (2) can be applied.

(5) $$\begin{array}{ccc} \sigma_{ct}\left(w\right) & \approx & \frac{1}{A_{C}}\sum_{k=1}^{N}P\left(w,\theta_{k},l_{e,k}\right) \end{array}$$

(6) $$\begin{array}{ccc} & \approx & \frac{1}{A_{C}}\sum_{i=1}^{9}\sum_{j=1}^{3}N_{T_{i},L_{j}}\tilde{P}\left(w,\theta_{i},l_{e,j}\right). \end{array}$$

Here, $N_{T_{i},L_{j}}$ denotes the number of fibres in *C* with $\theta_{k}\in T_{i}$ and $l_{e,k}\in L_{j}$, k∈{1,⋯,N}, i∈{1,⋯,9}, j∈{1,2,3}. $\tilde{P}$ is a prediction of *P* by the median curve or by the trilinear model. We denote the prediction based on $\tilde{P}=\dot{P}$ by $\sigma_{ct}^{med}\left(w\right)$ and the prediction based on $\tilde{P}=P_{tri}$ by $\sigma_{ct}^{tri}\left(w\right)$.

### 2.6. Stochastic Fibre Model

In the following, we introduce a stochastic fibre model to generate 3D fibre systems of virtual specimens. Their tensile behaviour can then be predicted with our stress prediction model.

The fibre system is modelled by a Boolean model [53] as follows: The positions of fibres are indicated by their midpoints, which are modelled by a Poisson point process. That is, the number *N* of fibre midpoints (x,y,z) in a given volume *V* follows a Poisson distribution with parameter λ·V, where λ>0 is the mean number of fibres per unit volume. Locations of fibre midpoints are drawn independently from a uniform distribution on the volume of interest.

The orientation of a fibre is independent of its location and can be described in spherical coordinates with co-latitude angle θ∈[0,π/2) and longitude angle φ∈[0,2π). Assuming the Z-axis to be the tension axis, θ corresponds to the inclination angle of the fibre with respect to the tension axis. The longitude angle φ does not influence the force contribution of a fibre. Hence, we consider φ to be uniformly distributed on [0,2π) such that the probability density function of the fibre orientation is a function p(θ) depending only on θ.

**Figure 5:**
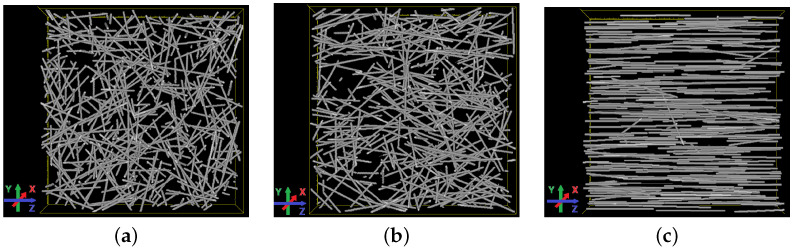
Realisations of the fibre model for different anisotropy parameters β. (**a**) β=1 (isotropic); (**b**) β=0.5; (**c**) β=0.01.

We assume that θ follows a one-parametric orientation distribution described by the p.d.f

(7) $$\begin{array}{c} p\left(\theta \right)=\frac{\beta \sin(\theta )}{{\left(1+\left(\beta^{2}-1\right)\cos^{2}\left(\theta \right)\right)}^{\frac{3}{2}}},\;\theta \in \left[0,\frac{\pi }{2}\right],\;\beta >0, \end{array}$$

see [50,54,55,56] for details and applications. The anisotropy parameter β controls the alignment of the fibres. For β=1, the fibres are isotropically oriented. For decreasing β, the fibres tend to be aligned along the Z-axis, see Figure 5. For β>1, the fibre orientations are concentrated in a plane. This case is not considered here.

For fitting the model to a given concrete sample, the characteristics λ and β can be obtained from μCT images, see [50,55,57]. Due to the low fibre volume fraction, we assume that overlap of fibres in the model is negligible. Hence, the fibre intensity λ can be computed from the fibre volume fraction $V_{V}$, the fibre length $l_{f}$ and cross-sectional area $A_{f}$ via

(8) $$\begin{array}{c} \lambda =\frac{V_{V}}{l_{f}A_{f}}. \end{array}$$

If a single-fibre segmentation is available, an estimate of the anisotropy parameter β can be determined from the sample $\theta_{1},\ldots ,\theta_{N}$ of inclination angles by using the maximum likelihood method as described in [54]. An estimate of β based on the method of moments is given by (see Appendix B)

(9) $$\begin{array}{cc} {\hat{\beta }}_{mom} & =\frac{N}{\sum_{i=1}^{N}\cos\left(\theta_{i}\right)}-1. \end{array}$$

The Boolean model described so far yields a stochastic fibre system in 3D. The prediction model outlined in Section 2.5 requires only inclination angles θ and embedded lengths $l_{e}$ of the fibres intersecting the crack plane *C*. Due to the spatial homogeneity of the model, *C* can be assumed to be contained in the (X,Y)-plane. In the Boolean model, fibre position and orientation are independent. Hence, $l_{e}$ and θ are independent. Thus, the joint p.d.f. of θ and $l_{e}$ for crack-crossing fibres fulfils $p_{c}\left(\theta ,l\right)=p_{c}\left(\theta \right)p_{l_{e}}\left(l\right)$ where $p_{c}\left(\theta \right)$ is the p.d.f. of inclination angles θ of fibres crossing the crack and $p_{l_{e}}\left(l\right)$ is the p.d.f. of $l_{e}$. The spatial homogeneity of the Boolean model implies that $l_{e}$ is uniformly distributed on $\left[0;\;l_{f}/2\right]$. The planar characteristics are related to the spatial characteristics as follows [49,50,53]

(10) $$\begin{array}{cc} p_{l_{e}}\left(l\right) & =\frac{2}{l_{f}},\;l\in \left[0;\;l_{f}/2\right] \end{array}$$

(11) $$\begin{array}{cc} p_{c}\left(\theta \right) & =(1+\beta )p(\theta )\cos(\theta ) \end{array}$$

(12) $$\begin{array}{cc} \lambda_{c} & =\frac{\lambda l_{f}}{1+\beta } \end{array}$$

where $\lambda_{c}$ is the expected number of fibres per unit area in *C*.

Note that the difference between (7) and (11) can be explained as follows [49,58]: The p.d.f. p(θ) takes every fibre in the reinforced concrete into account whereas $p_{c}\left(\theta \right)$ accounts only for fibres intersecting the crack plane. A fibre is more likely to hit the crack plane if its inclination angle θ is small. The assumption that the distribution $p_{c}\left(\theta \right)$ is equal to p(θ) is a common misunderstanding that leads to incorrect interpretations of measurement results (see [22,31,59]).

If a single-fibre segmentation of fibres in a crack is available, an estimate of the anisotropy parameter β can be determined from the sample $\theta_{c,1},\cdots ,\theta_{c,N}$ of inclination angles of crack-crossing fibres by using a modified version of the maximum likelihood estimator given in [54]. An estimator of β based on the method of moments is given by

(13) $$\begin{array}{cc} {\hat{\beta }}_{c,mom} & =\frac{N}{\sum_{i=1}^{N}\cos^{2}\left(\theta_{c,i}\right)}-1, \end{array}$$

see Appendix B for details.

The fibre orientation coefficient $\eta_{\theta }$ is a widely established characteristic for the distribution of fibre orientation in a crack [22,31,33,50,60,61,62]. $\eta_{\theta }$ is the second moment of the angular deviation of the crack-crossing fibre from the tension axis. Here, it is possible to calculate $\eta_{\theta }$ in closed form:

(14) $$\begin{array}{cc} \eta_{\theta } & =\int_{0}^{\frac{\pi }{2}}p_{c}\left(\theta \right)\cos^{2}\left(\theta \right)\;d\theta =\frac{1}{1+\beta }=\frac{\lambda_{c}}{\lambda l_{f}} \end{array}$$

For an isotropic fibre orientation (β=1), which is assumed in several studies [19,22,31,34], it follows that $\lambda_{c}=\frac{1}{2}\lambda l_{f}$, $p_{c}\left(\theta \right)=2\sin\left(\theta \right)\cos\left(\theta \right)=\sin\left(2\theta \right)$ and $\eta_{\theta }=\frac{1}{2}$. In the extreme case that all fibres are aligned along the tension axis (β=0), it follows that $\lambda_{c}=\lambda l_{f}$, and $\eta_{\theta }=1$.

## 3. Results and Discussion

### 3.1. Image Analysis

**Figure 6:**
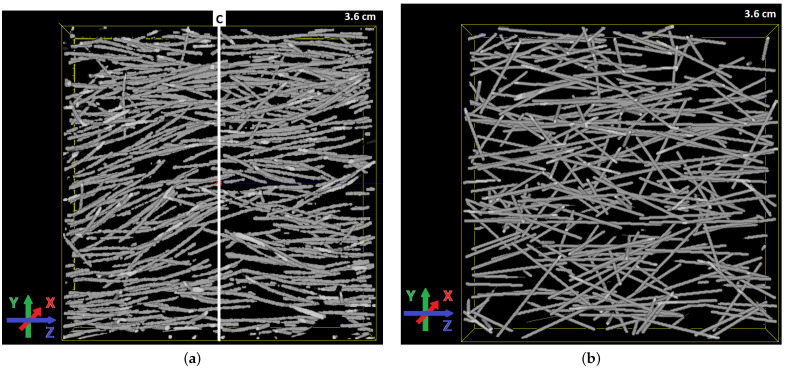
Volume rendering of fibres in the crack area (**a**) and a fitted fibre system (**b**). The subvolume is of dimension 3.6 cm${}^{2}$× 0.45 cm. The position of *C* in (**a**) is indicated by a solid white line.

The crack observed in the imaged sample is approximated by a plane whose position is identified in the CT scan, see Figure 6a. Among the segmented fibres, N=598 cross the crack plane. Inclination angles of the individual fibres are determined by using partial second derivatives as described in our former study [13]. Fibre lengths can be measured by the maximal Feret diameter [63]. Note that the Feret diameter of a particle is defined as the minimal distance between two parallel planes that are orthogonal to a specified direction and enclose the particle. The maximal Feret diameter is obtained by maximization over all directions. By intersection with the crack plane, each fibre is split into two segments. The embedded length $l_{e}$ is the maximal Feret diameter of the shorter one of these segments. From the inclination angles $\theta_{c,i}$, we obtain ${\hat{\beta }}_{c,mom}=0.22$.

By Equation (12) with β = 0.22 the expected number of fibres in the cross section is $A_{C}\cdot \lambda_{c}=834$. The observed number *N* = 598 is well below that value. There are several possible explanations. In [13], the fibre content at the crack location was observed to be about 5% lower than the theoretical value of $V_{V}$ = 0.02 (see [13] Figure 12g at the approximate crack position in Slice 641). Additionally, according to the master datasheet of the fibres used, both, the diameter $d_{f}$ and the fibre length $l_{f}$ can deviate by 10%.

**Figure 7:**
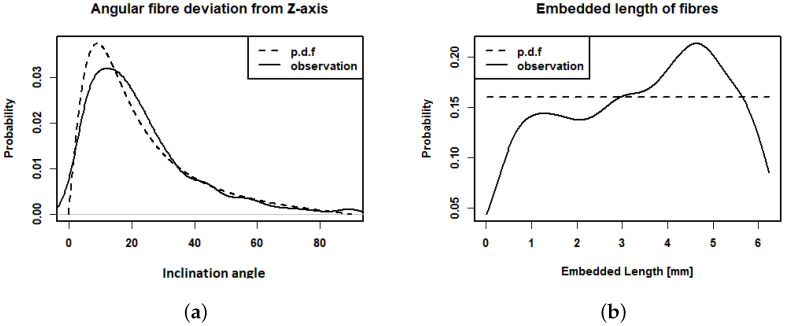
Observed inclination angle (**a**) and embedded length (**b**) of the fibres intersecting the crack (solid lines) and the corresponding fitted p.d.f. $p_{c}\left(\theta \right)$ with β=0.22 and $p_{l_{e}}\left(l\right)$ (dotted lines).

In Figure 7, the distributions of observed inclination angles and embedded lengths are compared with the fitted p.d.f.s $p_{c}\left(\theta \right)$ (with β=0.22) and $p_{l_{e}}\left(l\right)$. The inclination angles are well fitted. A chi-square goodness-of-fit test does not reject the hypothesis that the inclination angles follow a distribution with p.d.f. $p_{c}\left(\theta \right)$ with β=0.22 (*p*-value = 0.1244). The distribution of the embedded length has an unexpected peak around 4.5 mm. The hypothesis that the embedded length are uniformly distributed is rejected by a Kolmogorov–Smirnov goodness-of-fit test (*p*-value < $10^{-4}$). A possible explanation is that short fibre segments (whose diameter in the μCT image corresponds to only 2 to 3 voxels) were missed. It should be noted that we are not aware of any practical way of estimating $l_{e}$ from a 2D slice as was also mentioned in [22]. A fibre system simulated as a realisation of the Boolean model with β=0.22, $l_{f}=12.5$ mm, $d_{f}=0.2$ mm, and $V_{V}=0.02$ is shown in Figure 6b.

### 3.2. Prediction of Tensile Stress

To predict the composite stress $\sigma_{ct}$ using Equation (6), we need $N_{T_{i},L_{j}}$, the number of crack-crossing fibres with $\left(\theta ,l_{e}\right)$ in class $(T_{i},L_{j}),\;i=1,\cdots ,9,\;j=1,2,3$. Based on the single-fibre segmentation, we derive $N_{T_{i},L_{j}}$ as given in Table 3.

Experimental tensile tests were carried out on three specimens as described in Section 2.3. The ultimate tensile stress and the corresponding strain of the experimental tensile curves are given in Table 4. The observed tensile curves are used to calibrate our prediction.

#### 3.2.1. Prediction of Tensile Stress Based on Median Curve

**Figure 8:**
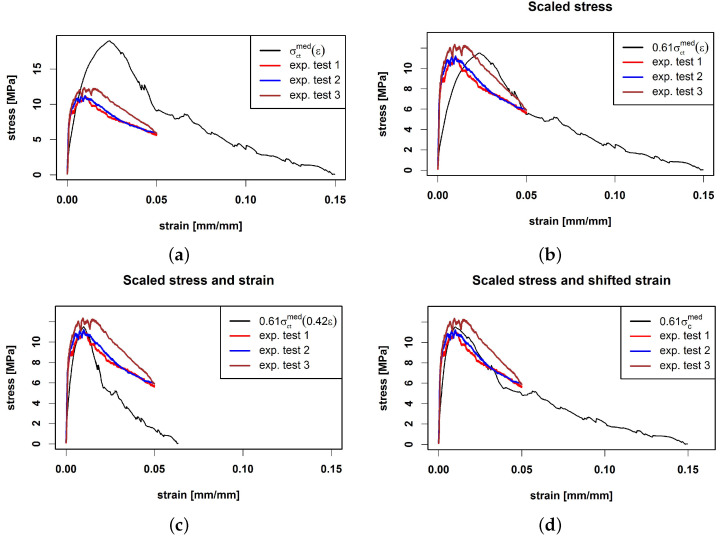
Prediction of composite stress $\sigma_{ct}\left(\varepsilon \right)$ by $\sigma_{ct}^{med}\left(\varepsilon \right)$ (**a**) compared with three experimental tensile curves. Scaled $\sigma_{ct}^{med}\left(\varepsilon \right)$ by stress scaling factor $S_{ult}^{\sigma }=0.61$ (**b**) and additionally scaled strain by strain scaling factor $S_{ult}^{\varepsilon }=0.42$ (**c**). (**d**) Prediction based on Equation (15).

Here, we use the median curve to predict $\sigma_{ct}$ by $\sigma_{ct}^{med}$. Figure 8a shows the prediction as well as the three experimental tensile curves. We see that the prediction overestimates the stress. The predicted ultimate tensile stress is 18.56 MPa which is reached at strain $\varepsilon_{ult}^{med}=0.0230$ [mm/mm].

To calibrate the curves, we follow [19,22,31,64] and introduce suitable rescaling factors to match characteristic stress-strain points (i.e., ultimate tensile stress $\sigma_{ct,ult}$ or yield stress $\sigma_{ct,el}$) of experiments and predictions.

In the first step, we scale $\sigma_{ct}^{med}$ by a stress scaling factor $S_{ult}^{\sigma }$ such that the predicted and mean experimental ultimate stress coincide, i.e., $S_{ult}^{\sigma }\sigma_{ct,ult}^{med}=\bar{\sigma_{ult,exp}}$. This is achieved for $S_{ult}^{\sigma }=0.61$, see Figure 8b. In the next step, the locations of the maxima have to be matched. To this end, we additionally scale the strain axis by a strain scaling factor $S_{ult}^{\varepsilon }$. The choice $S_{ult}^{\varepsilon }=0.42$ yields $S_{ult}^{\varepsilon }\varepsilon_{ult}^{med}=\bar{\varepsilon_{ult,exp}}$ such that the maxima are closer together (see Figure 8c); however, the slope of the curve after the maximum does not fit the experimental curves. A remedy is to restrict scaling of the strain axis to the region prior to the maximum, that is,

(15) $$\begin{array}{cc} \sigma_{ct}^{med,scale}\left(\varepsilon \right)\; & =\left\{\begin{array}{cc} \sigma_{ct}^{med}\left(\frac{\varepsilon }{S_{ult}^{\varepsilon }}\right) & \varepsilon \in \left[0,\bar{\varepsilon_{ult,exp}}\right]\\ \sigma_{ct}^{med}\left(\varepsilon_{ult}^{med}+\frac{\varepsilon_{tot}^{med}-\varepsilon_{ult}^{med}}{\varepsilon_{tot}^{med}-\bar{\varepsilon_{ult,exp}}}\cdot \left(\varepsilon -\bar{\varepsilon_{ult,exp}}\right)\right) & \varepsilon \in \left(\bar{\varepsilon_{ult,exp}},\varepsilon_{tot}^{med}\right], \end{array}\right. \end{array}$$

where $\varepsilon_{tot}^{med}$ is the last available strain value in the median curve. This way, both, the maximum location and the stress at the final point $\varepsilon_{tot}^{med}$ are matched. The result shown in Figure 8d indicates that the slope after the ultimate tensile stress is reduced due to the fixation of $\varepsilon_{tot}^{med}$.

#### 3.2.2. Prediction of Tensile Stress Based on the Trilinear Model

**Figure 9:**
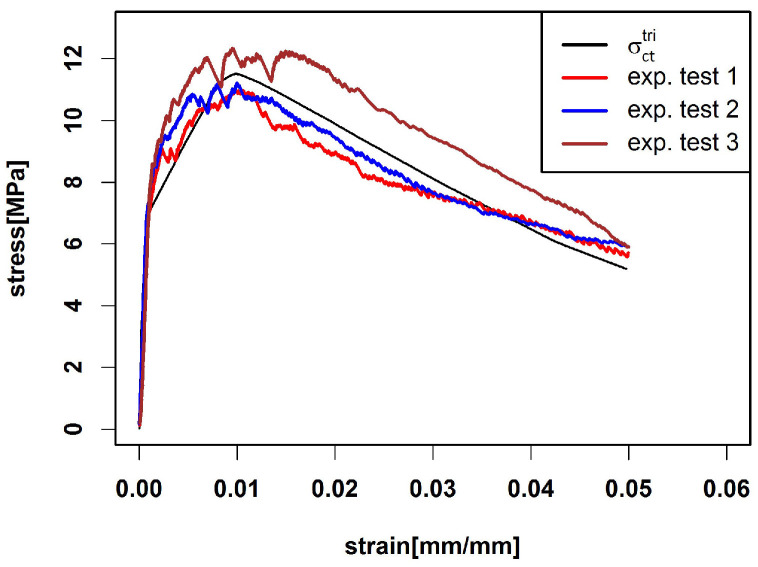
Prediction of composite stress $\sigma_{ct}\left(\varepsilon \right)$ by $\sigma_{ct}^{tri}\left(\varepsilon \right)$ based on the trilinear model.

Here, we use model (2) to predict $\sigma_{ct}$ by $\sigma_{ct}^{tri}$. As for the median curve we need to modify the prediction. Instead of piecewise scaling equivalent to Equation (15), we recompute (2) with new values for slip and force given by

(16) $$\begin{array}{cc} s_{el,med}^{\circ }\left(\theta ,l_{e}\right) & =0.072\cdot s_{el,med}\left(\theta ,l_{e}\right) \end{array}$$

(17) $$\begin{array}{cc} s_{ult,med}^{\circ }\left(\theta ,l_{e}\right) & =0.36\cdot s_{ult,med}\left(\theta ,l_{e}\right) \end{array}$$

(18) $$\begin{array}{cc} s_{tot,med}^{\circ }\left(\theta ,l_{e}\right) & =s_{tot,med}\left(\theta ,l_{e}\right) \end{array}$$

(19) $$\begin{array}{cc} P_{el,med}^{\circ }\left(\theta ,l_{e}\right) & =0.66\cdot P_{el,med}\left(\theta ,l_{e}\right) \end{array}$$

(20) $$\begin{array}{cc} P_{ult,med}^{\circ }\left(\theta ,l_{e}\right) & =0.66\cdot P_{ult,med}\left(\theta ,l_{e}\right) \end{array}$$

where $s_{el,med}\left(\theta ,l_{e}\right),s_{ult,med}\left(\theta ,l_{e}\right),s_{tot,med}\left(\theta ,l_{e}\right),P_{el,med}\left(\theta ,l_{e}\right),P_{ult,med}\left(\theta ,l_{e}\right)$ are given in Table Table A1. The new parameters ($p_{1}^{\circ },p_{2}^{\circ },p_{3}^{\circ },r_{1}^{\circ },r_{2}^{\circ }$) are calculated as in Appendix A2. The prediction is given in Figure 9. The factors are chosen such that $\sigma_{ct,ult}^{tri}=\bar{\sigma_{ult,exp}}$ and $\varepsilon_{ct,ult}^{tri}=\bar{\varepsilon_{ult,exp}}$.

In the interval between yield and ultimate stress, the predicted curve is straighter than the experimental stress curves, which can be explained by the use of a linear model. Furthermore, the maximal peak is more pronounced than in the experimental tests. This may be due to the replacement of the individual single-fibre pull-out forces $P_{i}$ by average forces $P_{tri}$ obtained from the trilinear model. To overcome this problem, we propose a model based on randomised single-fibre contributions in the following section.

#### 3.2.3. Prediction of Tensile Curves Based on Randomised Trilinear Model

In order to model the random variations in the tensile curves, we consider $s_{ult,med}$ as random following a uniform distribution and add normally distributed residuals to the yield and the ultimate force. This way, individual functions $\tilde{P}$ are simulated for each fibre in the crack. The range of values of the required random variables is inferred from the single-fibre pull-out experiments.

Prediction study part 1: Stress prediction with randomised strain shifts

We define the interval

(21) $$\begin{array}{cc} I_{ult}\left(\theta ,l_{e}\right) & =\left[\min_{i=1,\cdots ,6}\left(s_{ult,i}\left(\theta ,l_{e}\right)\right);\;\max_{i=1,\cdots ,6}\left(s_{ult,i}\left(\theta ,l_{e}\right)\right)\right] \end{array}$$

and choose

(22) $$\begin{array}{cc} s_{ult,med}^{*}\left(\theta ,l_{e}\right) & ∼U(I_{ult}\left(\theta ,l_{e}\right)) \end{array}$$

(23) $$\begin{array}{cc} r_{P_{el,med}}^{*}\left(\theta ,l_{e}\right) & ∼N(0,{\mathrm{sd}}_{P_{el,med}}^{2}\left(\theta ,l_{e}\right)) \end{array}$$

(24) $$\begin{array}{cc} r_{P_{ult,med}}^{*}\left(\theta ,l_{e}\right) & ∼N(0,{\mathrm{sd}}_{P_{ult,med}}^{2}\left(\theta ,l_{e}\right)), \end{array}$$

where the minimum and maximum values of $s_{ult,i}$ and the sample standard deviations ${\mathrm{sd}}_{P_{el,med}}$ and ${\mathrm{sd}}_{P_{ult,med}}$ for each combination $\left(\theta ,l_{e}\right)$ are given in Appendix A, Table A1. Then, we recompute the trilinear curve with

(25) $$\begin{array}{cc} s_{el,med}^{\circ }\left(\theta ,l_{e}\right) & =0.072\cdot s_{el,med}\left(\theta ,l_{e}\right) \end{array}$$

(26) $$\begin{array}{cc} s_{ult,med}^{\circ }\left(\theta ,l_{e}\right) & =0.36\cdot s_{ult,med}^{*}\left(\theta ,l_{e}\right) \end{array}$$

(27) $$\begin{array}{cc} s_{tot,med}^{\circ }\left(\theta ,l_{e}\right) & =s_{tot,med}\left(\theta ,l_{e}\right) \end{array}$$

(28) $$\begin{array}{cc} P_{el,med}^{\circ }\left(\theta ,l_{e}\right) & =0.68\cdot P_{el,med}\left(\theta ,l_{e}\right)+r_{P_{el,med}}^{*}\left(\theta ,l_{e}\right) \end{array}$$

(29) $$\begin{array}{cc} P_{ult,med}^{\circ }\left(\theta ,l_{e}\right) & =0.68\cdot P_{ult,med}\left(\theta ,l_{e}\right)+r_{P_{ult,med}}^{*}\left(\theta ,l_{e}\right). \end{array}$$

**Figure 10:**
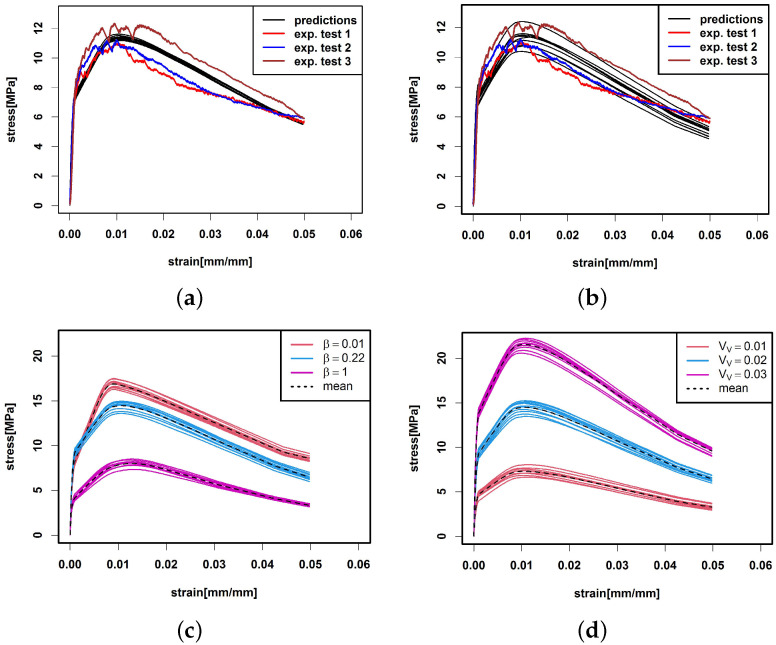
Prediction of *M* = 10 tensile curves (**a**) based on the CT scanned fibre system summarised in Table 3, (**b**) tensile prediction of 10 virtual specimens with fibre system generated by the stochastic fibre model from Section 2.6 with input parameters determined from the scanned fibre system. The predictions are compared with the three experimental tensile curves. (**c**) and (**d**) Tensile prediction of 10 virtual specimens with varied parameters. (**c**)β=0.01,0.22,1 is varied and $V_{V}$ is fixed. (**d**) β is fixed and $V_{V}=0.01,0.02,0.03$ is varied. The mean of the tensile predictions per varied parameter is given as a dotted line.

M=10 predictions based on the fibre system observed in the CT image are shown in Figure 10a. The predictions reproduce the stress profile better than the deterministic predictions.

Prediction study part 2: Stress prediction of virtual specimens with varying production parameters

We use the stochastic fibre model from Section 2.6 to generate fibre systems of virtual specimens. The model parameters are determined from the crack-crossing fibres of the scanned specimen. From each model realization, we derive the values of $\left(\theta ,l_{e}\right)$ for all fibres intersecting a virtual crack plane *C*. Figure 10b shows a tensile prediction of ten virtual fibre systems when using the randomized prediction scheme described above.

Predictions for virtual samples with modified orientation distribution and volume fraction $V_{V}$ are shown in Figure 10. Note that the fibre parameters $d_{f}$ and $l_{f}$ were not varied since the single-fibre pull-out tests were restricted to fibres with $d_{f}=0.2$ mm and $l_{f}=12.5$ mm. Figure 10c shows that a fibre orientation along the tensile axis increases the tensile stress compared to an isotropic fibre orientation. This is in accordance with [22,31]. In particular, this plot shows that the common assumptions of completely aligned or isotropic fibres result in quite different predictions. Figure 10d reveals that the influence of the volume fraction also behaves as expected: Fewer fibres decrease and more fibres increase the tensile stress.

## 4. Conclusions

In this study, we present a prediction model for tensile behaviour of UHPFRC-specimens based on statistical information on the fibre system and extensive single-fibre pull-out tests. The model is calibrated by comparing the prediction to the results of experimental uniaxial tensile tests. We introduced a stochastic fibre model that allows for the generation of fibre systems of virtual specimens, which can be used for the prediction of the tensile behaviour. Through experimental and theoretical investigations, the following conclusions are drawn:

- The fibre system in the concrete is modelled by a Boolean model of straight cylinders. The fibre orientation distribution is represented by a one-parametric distribution family. Its parameter β controls the anisotropy of the fibre orientation. Both, the case of total alignment and isotropy are included in the model as special cases. The widely used reference value $\eta_{\theta }$ (fibre orientation factor) can be calculated directly from β. In particular, the relationship of fibre orientation of the full 3D fibre system and of crack-crossing fibres is examined.
- We emphasised the difference between the p.d.f. for the fibre orientation of all fibres in the reinforced concrete and the p.d.f. for the fibre orientation of fibres intersecting a crack plane. The assumption that the p.d.f.s are equal is a common misunderstanding that leads to incorrect interpretations of measurement results (see [22,31,59]).
- Estimators for β are presented and analysed. We recommend the method of moments estimator ${\hat{\beta }}_{c,mom}$. It estimates accurately, no numerical method is required (such as a gradient descent algorithm in [55]) and only the fibre angles in 2D cross-sections are needed.
- In contrast to 2D imaging methods, μCT allows for a determination of the embedded length of fibres in cracks since the whole 3D fibre system is observable. In particular, this allows for the validation of the fundamental assumption of a uniformly distributed embedded length $l_{e}$ for the widespread tension-softening model in [16]. Furthermore, using μCT overcomes the accuracy disadvantage of fibre orientation estimation in 2D sections as discussed in [22].
- The presented prediction model uses the stochastic fibre model combined with a statistical analysis and stochastic modelling of the single-fibre pull-out tests. Predictions are calibrated by using experimental tensile curves. Running the prediction on virtual specimens with varied production parameters led to reasonable stress–strain curves. More research with additional samples is needed to validate the model and investigate its robustness to changes in the production parameters.

## Figures and Tables

**Table 1:** Constituents (given in grams per litre), bending tensile strength and compression strength of the UHPC-mixture used in the tests.

Cement CEM I 52.5 R SR3- NA (Sulfo 5R)	825 g/L
Quarz Sand 0.125/0.5 Haltern	975 g/L
Quarz Flour-MILLISIL-W12	200 g/L
Silica fume: Sika Silicoll P uncompacted	175 g/L
Water	179 g/L
PCE-plasticizer—Sika Viscocrete 2810	30.25 g/L
Strain at uniaxial tensile strength ($\varepsilon_{r}$)	0.00087 [mm/mm]
Uniaxial tensile strength	6.4 MPa
Bending tensile strength	13.77 MPa
Compression strength	150.2 MPa

**Table 2:** Binning of embedded length $l_{e}$ and inclination angle θ.

Class	Interval	$P(w,\theta ,l_{e})$
$T_{1}$	$\theta \in [\;0^{\circ };5^{\circ })$	$P(w,\;0^{\circ },l_{e})$
$T_{2}$	$\theta \in [5^{\circ };15^{\circ })$	$P(w,10^{\circ },l_{e})$
⋮	⋮	⋮
$T_{9}$	$\theta \in [75^{\circ };90^{\circ }]$	$P(w,80^{\circ },l_{e})$
$L_{1}$	$l_{e}\in$ [0 mm; 2.5 mm)	P(w,θ,2.08)
$L_{2}$	$l_{e}\in$ [2.5 mm; 5 mm)	P(w,θ,4.17)
$L_{3}$	$l_{e}\in$ [5 mm; 6.25 mm)	P(w,θ,6.25)

**Table 3:** Number of fibres in the $\left(\theta ,l_{e}\right)$ bins as obtained from the single-fibre segmentation of the μCT image.

	$T_{i}$	$0^{\circ }$	$10^{\circ }$	$20^{\circ }$	$30^{\circ }$	$40^{\circ }$	$50^{\circ }$	$60^{\circ }$	$70^{\circ }$	$80^{\circ }$
$L_{j}$										
$l_{f}/2$		10	52	39	14	6	6	3	1	0
$l_{f}/3$		20	92	91	35	20	9	6	1	1
$l_{f}/6$		14	48	44	34	16	14	8	6	8

**Table 4:** Ultimate tensile stress and the corresponding tensile strain of three uniaxially loaded specimens.

	1	2	3	Mean
$\sigma_{ult}^{exp}$ [MPa]	10.97	11.21	12.34	$\bar{\sigma_{ult,exp}}$ = 11.51
$\varepsilon_{ult}^{exp}$ [mm/mm]	0.01064	0.0010	0.0095	$\bar{\varepsilon_{ult,exp}}$ = 0.0070

## Abbreviations

The following abbreviations are used in this manuscript:

UHPFRC	ultra-high performance fibre-reinforced concrete
μCT	micro-computed tomography
p.d.f.	probability density function
$l_{f}$	fibre length
$d_{f}$	fibre diameter
$A_{f}$	fibre cross-section
$V_{V}$	fibre volume fraction
*L*	tensioned length
*F*	force
*A*	area of the specimen cross-section
*C*	planar crack
$A_{C}$	area of crack *C*
*w*	crack width
*s*	slip
θ	inclination angle
$l_{e},l_{e}$	embedded length in single fibre pull-out tests, in cracked UHPFRC
$s_{el}\left(\theta ,l_{e}\right)$	slip value of $P_{el}\left(\theta ,l_{e}\right)$
$s_{ult}\left(\theta ,l_{e}\right)$	slip value of $P_{ult}\left(\theta ,l_{e}\right)$
$s_{tot}\left(\theta ,l_{e}\right)$	last slip value
$s_{el,med}\left(\theta ,l_{e}\right)$	median of $s_{el}\left(\theta ,l_{e}\right)$
$s_{ult,med}\left(\theta ,l_{e}\right)$	median of $s_{ult}\left(\theta ,l_{e}\right)$
$s_{tot,med}\left(\theta ,l_{e}\right)$	median of $s_{tot}\left(\theta ,l_{e}\right)$
$P(s,\theta ,l_{e})$	single-fibre pull-out force
$P_{el}\left(\theta ,l_{e}\right)$	tensile force at the end of the linear phase
$P_{ult}\left(\theta ,l_{e}\right)$	ultimate force
$\dot{P}$	points-wise median curve
$P_{tri}$	trilinear curve
$P_{el,med}\left(\theta ,l_{e}\right)$	median of the six fibre pull-out forces $P_{el}\left(\theta ,l_{e}\right)$
$P_{ult,med}\left(\theta ,l_{e}\right)$	median of $P_{ult}\left(\theta ,l_{e}\right)$
$p_{i},r_{j}$	parameters of $P_{tri}$, i=1,2,3,j=1,2
$T_{i},L_{j}$	classes for θ, $l_{e}$, i=1,⋯,9,j=1,2,3
$N_{T_{i},L_{j}}$	number of fibres with $\left(\theta ,l_{e}\right)\in \left(T_{i},L_{j}\right)$ in *C*
ε	strain
$\varepsilon_{r}$	crack strain
σ	uniaxial stress
$\sigma^{exp},\varepsilon^{exp}$	uniaxial stress, strain from experimental tensile curves
$\bar{\sigma_{ult,exp}},\bar{\varepsilon_{ult,exp}}$	mean values of $\sigma^{exp},\varepsilon^{exp}$
$\sigma_{ct}$	composite stress resp. mean resistance force per unit area
$\sigma_{ct}^{med},\sigma_{ct}^{tri}$	prediction of $\sigma_{ct}$ based on $\dot{P}$ and $P_{tri}$, respectively
$\sigma_{ct}^{med,scale}$	scaled version of $\sigma_{ct}^{med}$
$S_{ult}^{\sigma },S_{ult}^{\varepsilon }$	stress, strain scaling factor
p(θ)	spatial p.d.f of θ
$p_{c}\left(\theta .l\right)$	joint p.d.f of θ and $l_{e}$ in *C*
$p_{c}\left(\theta \right)$	p.d.f of θ in *C*
$p_{l_{e}}\left(l\right)$	p.d.f of $l_{e}$ in *C*
$\lambda ,\lambda_{c}$	mean of fibres per unit volume, per unit area in *C*
β	anisotropy parameter
${\hat{\beta }}_{mom}$	method of moments estimator of β based on 3D fibre system
${\hat{\beta }}_{c,mom}$	method of moments estimator of β based on fibres crossing *C*
U(I)	uniform distribution on interval *I*
$N(a,b^{2})$	normal distribution with mean *a* and variance $b^{2}$
$I_{ult}\left(\theta ,l_{e}\right)$	interval giving the range of $s_{ult,med}\left(\theta ,l_{e}\right)$ over all θ but a fixed $l_{e}$
$s_{ult,med}^{*}\left(\theta ,l_{e}\right)$	random value based on interval $I_{ult}\left(\theta ,l_{e}\right)$
${\mathrm{sd}}_{P_{ult,med}}\left(\theta ,l_{e}\right)$	sample standard deviations of $P_{ult,med}\left(\theta ,l_{e}\right)$
${\mathrm{sd}}_{P_{el,med}}\left(\theta ,l_{e}\right)$	sample standard deviations of $P_{el,med}\left(\theta ,l_{e}\right)$
$r_{P_{ult,med}}^{*}\left(\theta ,l_{e}\right)$	random values based on ${\mathrm{sd}}_{P_{ult,med}}\left(\theta ,l_{e}\right)$
$r_{P_{el,med}}^{*}\left(\theta ,l_{e}\right)$	random values based on ${\mathrm{sd}}_{P_{el,med}}\left(\theta ,l_{e}\right)$

## Appendix A

**Table A1:** Median values $s_{el,med}\left(\theta ,l_{e}\right),P_{el,med}\left(\theta ,l_{e}\right)$ (standard deviations given in brackets)$,s_{ult,med}\left(\theta ,l_{e}\right)$ (minimum and maximum values given in brackets)$,P_{ult,med}\left(\theta ,l_{e}\right)$ (standard deviations given in brackets)$,s_{tot,med}\left(\theta ,l_{e}\right)$. No values for ${\mathrm{sd}}_{P_{el,med}}$ and ${\mathrm{sd}}_{P_{ult,med}}$ were determined for the combination $\left(\theta ,l_{e}\right)=\left(80^{\circ },l_{f}/6\right)$, as only one single-fibre pull-out curve could be observed.

$\left(\theta ,l_{e}\right)$	$s_{\mathrm{el},\mathrm{med}}$	$P_{\mathrm{el},\mathrm{med}}$	$s_{\mathrm{ult},\mathrm{med}}$	$P_{\mathrm{ult},\mathrm{med}}$	$s_{\mathrm{tot},\mathrm{med}}$
	[mm]	[N]	[mm]	[N]	[mm]
$\left(0^{\circ },l_{f}/2\right)$	0.50	39.67 (32.73)	0.97 (0.76, 1.77)	47.52 (27.82)	5.68
$\left(0^{\circ },l_{f}/3\right)$	0.03	7.21 (18.80)	0.89 (0.69, 1.04)	53.30 ( 9.80)	4.82
$\left(0^{\circ },l_{f}/6\right)$	0.08	6.50 (13.70)	0.94 (0.46, 1.09)	23.82 (12.95)	1.82
$\left(10^{\circ },l_{f}/2\right)$	0.37	56.56 (15.69)	1.12 (0.42, 3.17)	87.52 (19.04)	5.15
$\left(10^{\circ },l_{f}/3\right)$	0.51	43.16 (10.30)	1.08 (0.93, 2.13)	52.85 (15.24)	3.82
$\left(10^{\circ },l_{f}/6\right)$	0.14	13.95 (15.16)	0.90 (0.60, 1.38)	27.62 (12.53)	2.02
$\left(20^{\circ },l_{f}/2\right)$	0.43	44.70 ( 8.59)	1.12 (0.65, 1.26)	66.68 (12.66)	5.77
$\left(20^{\circ },l_{f}/3\right)$	0.06	13.30 ( 5.93)	0.91 (0.77, 1.00)	39.86 (10.06)	3.46
$\left(20^{\circ },l_{f}/6\right)$	0.25	21.00 ( 4.49)	1.10 (0.73, 1.23)	24.10 (14.07)	1.67
$\left(30^{\circ },l_{f}/2\right)$	0.23	30.80 ( 5.62)	0.99 (0.66, 1.43)	56.90 (10.71)	4.91
$\left(30^{\circ },l_{f}/3\right)$	0.37	40.00 (18.23)	1.18 (0.63, 1.42)	52.74 (10.53)	2.61
$\left(30^{\circ },l_{f}/6\right)$	0.20	23.74 ( 9.09)	0.93 (0.83, 1.06)	44.70 ( 8.77)	1.70
$\left(40^{\circ },l_{f}/2\right)$	0.52	50.75 (14.04)	1.13 (0.73, 2.72)	71.35 (14.51)	4.99
$\left(40^{\circ },l_{f}/3\right)$	0.03	11.14 ( 6.63)	1.07 (0.66, 4.13)	63.52 (22.80)	3.45
$\left(40^{\circ },l_{f}/6\right)$	0.04	6.20 ( 8.70)	0.65 (0.28, 1.01)	23.78 (11.54)	1.42
$\left(50^{\circ },l_{f}/2\right)$	0.04	11.09 (14.00)	1.57 (1.38, 1.74)	53.34 (16.30)	4.59
$\left(50^{\circ },l_{f}/3\right)$	0.07	10.00 ( 7.70)	1.30 (0.97, 1.92)	44.85 (15.97)	2.48
$\left(50^{\circ },l_{f}/6\right)$	0.06	10.00 (27.88)	0.48 (0.35, 0.76)	25.41 ( 6.46)	0.88
$\left(60^{\circ },l_{f}/2\right)$	0.10	14.20 ( 6.18)	1.40 (1.17, 1.60)	68.25 (13.00)	4.96
$\left(60^{\circ },l_{f}/3\right)$	0.06	13.50 (44.59)	1.39 (1.33, 2.96)	56.71 (50.63)	2.60
$\left(60^{\circ },l_{f}/6\right)$	0.11	14.80 ( 5.22)	0.96 (0.63, 1.01)	36.79 ( 7.57)	1.23
$\left(70^{\circ },l_{f}/2\right)$	0.07	11.90 (18.93)	1.77 (1.64, 2.07)	59.29 (10.08)	4.16
$\left(70^{\circ },l_{f}/3\right)$	0.11	8.90 ( 3.87)	1.65 (0.84, 2.19)	44.99 (12.17)	2.51
$\left(70^{\circ },l_{f}/6\right)$	0.08	8.52 ( 3.52)	0.67 (0.38, 1.50)	20.73 ( 5.80)	1.11
$\left(80^{\circ },l_{f}/2\right)$	0.06	9.50 ( 5.24)	2.39 (1.17, 3.13)	54.94 (22.78)	3.77
$\left(80^{\circ },l_{f}/3\right)$	0.53	6.50 ( 2.81)	0.54 (0.02, 1.31)	7.27 ( 7.05)	0.69
$\left(80^{\circ },l_{f}/6\right)$	0.00	4.54 (-)	0.13 (0.13, 0.13)	4.55 (-)	0.37

**Table A2:** Parameter values for $P_{tri}\left(w,\theta ,l_{e}\right)$. No value for $p_{1}$ was determined for the combination $\left(\theta ,l_{e}\right)=\left(80^{\circ },l_{f}/6\right)$, as no phase I could be observed.

$\left(\theta ,l_{e}\right)$	$p_{1}$	$p_{2}$	$p_{3}$	$r_{1}$	$r_{2}$
$\left(0^{\circ },l_{f}/2\right)$	79.34	16.30	−26.83	31.78	46.58
$\left(0^{\circ },l_{f}/3\right)$	250.41	53.42	−31.58	5.67	49.68
$\left(0^{\circ },l_{f}/6\right)$	80.25	20.15	−35.99	4.87	21.61
$\left(10^{\circ },l_{f}/2\right)$	152.59	41.31	−57.37	41.25	94.03
$\left(10^{\circ },l_{f}/3\right)$	84.04	17.14	−41.81	34.35	56.04
$\left(10^{\circ },l_{f}/6\right)$	103.13	17.84	−34.24	11.54	24.07
$\left(20^{\circ },l_{f}/2\right)$	105.09	31.73	−40.63	31.20	71.22
$\left(20^{\circ },l_{f}/3\right)$	207.58	31.36	−29.84	11.29	37.08
$\left(20^{\circ },l_{f}/6\right)$	85.4	3.61	−58.37	20.11	29.89
$\left(30^{\circ },l_{f}/2\right)$	131.09	34.77	−35.44	22.63	56.39
$\left(30^{\circ },l_{f}/3\right)$	107.83	15.81	−66.17	34.13	63.52
$\left(30^{\circ },l_{f}/6\right)$	119.58	28.52	−74.70	18.08	39.54
$\left(40^{\circ },l_{f}/2\right)$	98.36	33.39	−48.13	33.52	77.35
$\left(40^{\circ },l_{f}/3\right)$	404.92	50.37	−54.20	9.75	67.05
$\left(40^{\circ },l_{f}/6\right)$	139.23	29.15	−30.17	4.90	10.66
$\left(50^{\circ },l_{f}/2\right)$	282.54	27.68	−49.61	10.00	75.59
$\left(50^{\circ },l_{f}/3\right)$	151.83	28.24	−69.47	8.14	63.08
$\left(50^{\circ },l_{f}/6\right)$	181.48	36.50	−41.19	7.99	−5.04
$\left(60^{\circ },l_{f}/2\right)$	136.65	41.59	−54.03	9.88	86.58
$\left(60^{\circ },l_{f}/3\right)$	242.58	32.42	−90.14	11.70	86.3
$\left(60^{\circ },l_{f}/6\right)$	133.94	25.89	−150.39	11.94	30.63
$\left(70^{\circ },l_{f}/2\right)$	175.64	27.91	−69.22	10.01	98.67
$\left(70^{\circ },l_{f}/3\right)$	83.08	23.36	−107.43	6.40	98.92
$\left(70^{\circ },l_{f}/6\right)$	109.05	20.72	−40.51	6.90	4.34
$\left(80^{\circ },l_{f}/2\right)$	152.08	19.49	−121.31	8.28	160.83
$\left(80^{\circ },l_{f}/3\right)$	12.28	122.75	−29.50	−58.49	−11.14
$\left(80^{\circ },l_{f}/6\right)$	-	0.08	-4.29	4.55	−4.27

The parameters where determined as follows

(A1) $$\begin{array}{cc} p_{1} & =\frac{P_{el,med}\left(\theta ,l_{e}\right)}{s_{el,med}\left(\theta ,l_{e}\right)} \end{array}$$

(A2) $$\begin{array}{cc} p_{2} & =\frac{P_{ult,med}\left(\theta ,l_{e}\right)-P_{el,med}\left(\theta ,l_{e}\right)}{s_{ult,med}\left(\theta ,l_{e}\right)-s_{el,med}\left(\theta ,l_{e}\right)} \end{array}$$

(A3) $$\begin{array}{cc} p_{3} & =\frac{P_{ult,med}\left(\theta ,l_{e}\right)}{s_{ult,med}\left(\theta ,l_{e}\right)-s_{tot,med}\left(\theta ,l_{e}\right)}, \end{array}$$

(A4) $$\begin{array}{cc} r_{1} & =P_{el,med}\left(\theta ,l_{e}\right)-p_{2}\cdot s_{el,med}\left(\theta ,l_{e}\right), \end{array}$$

(A5) $$\begin{array}{cc} r_{2} & =P_{ult,med}\left(\theta ,l_{e}\right)-p_{3}\cdot s_{ult,med}\left(\theta ,l_{e}\right). \end{array}$$

## Appendix B

We consider a fibre system as a realisation of a set of random line segments. The random angular deviation between a line segment and the Z-axis is described by its inclination angle θ. A random inclination angle in space is denoted by Θ and a random inclination angle of a fibre intersecting the (X,Y)-plane by $\Theta_{c}$. Θ follows a distribution with density

(A6) $$\begin{array}{c} p\left(\theta \right)=\frac{\beta \sin(\theta )}{{\left(1+\left(\beta^{2}-1\right)\cos^{2}\left(\theta \right)\right)}^{\frac{3}{2}}},\;\theta \in \left[0,\frac{\pi }{2}\right],\;\beta >0. \end{array}$$

Then, $\Theta_{c}$ follows a distribution with density [49]

(A7) $$\begin{array}{cc} p_{c}\left(\theta \right) & =\left(1+\beta \right)p\left(\theta \right)\cos\left(\theta \right),\;\theta \in \left[0,\frac{\pi }{2}\right],\;\beta >0. \end{array}$$

The difference between p(θ) and $p_{c}\left(\theta \right)$ arises from the fact that $p_{c}\left(\theta \right)$ only considers the fibres intersecting the (X,Y)-plane, whereas p(θ) considers every fibre of the fibre system [49]. In a realisation with *N* line segments, we denote a sample of spatial inclination angles by $\theta_{1},\cdots ,\theta_{N}$ and a sample of inclination angles of fibres intersecting the (X,Y)-plane by $\theta_{c,1},\cdots ,\theta_{c,M}$, M≤N.

### Method of Moments Estimators ${\hat{\beta }}_{\mathrm{mom}}$, ${\hat{\beta }}_{\mathrm{c, mom}}$ for β

The method of moments (mom) is based on the approximation of sample moments with theoretical moments (via the law of large numbers (LLN)). If the theoretical moments are functions of a parameter, the sample moments are functions of the parameter’s estimate. Here, the parameter of interest is the anisotropy parameter β. Assume that $\Theta_{1},\cdots ,\Theta_{N}$ i.i.d. with density *p* such that for suitable function *g* we have $E\left(\Theta_{i}^{k}\right)=g_{k}\left(\beta \right)<\infty$. Then an estimator ${\hat{\beta }}_{mom}$ can be obtained as solution of ${\bar{\Theta^{k}}}_{N}=\frac{1}{N}\sum_{i=1}^{N}\Theta_{i}^{k}=g_{k}\left({\hat{\beta }}_{mom}\right).$ We apply the cosine to the angles in the following. Thus, $\cos\left(\Theta_{1}\right),\cdots ,\cos\left(\Theta_{N}\right)$ are i.i.d. with

(A8) $$\begin{array}{cc} E(\cos\left(\Theta_{i}\right)) & =\int_{0}^{\frac{\pi }{2}}\cos\left(\theta \right)p\left(\theta \right)\;d\theta =\frac{1}{1+\beta } \end{array}$$

and $g\left(\beta \right)=\frac{1}{1+\beta },\beta >0$. Therefore,

(A9) $$\begin{array}{cc} {\hat{\beta }}_{mom} & =\frac{1}{{\bar{\cos(\Theta )}}_{N}}-1 \end{array}$$

with ${\bar{\cos(\Theta )}}_{N}=\frac{1}{N}\sum_{i=1}^{N}\cos\left(\Theta_{i}\right)$.

For $\Theta_{c,1},\cdots ,\Theta_{c,M}$ i.i.d. it follows analogously

(A10) $$\begin{array}{cc} E(\cos^{2}\left(\Theta_{c,i}\right))\; & =\int_{0}^{\frac{\pi }{2}}\cos^{2}\left(\theta \right)p_{c}\left(\theta \right)\;d\theta =\left(1+\beta \right)\int_{0}^{\frac{\pi }{2}}\cos^{3}\left(\theta \right)p\left(\theta \right)\;d\theta =\frac{1}{1+\beta } \end{array}$$

Therefore,

(A11) $$\begin{array}{cc} {\hat{\beta }}_{c,mom} & =\frac{1}{{\bar{\cos^{2}\left(\Theta_{c}\right)}}_{M}}-1. \end{array}$$
